# Intelligent Identification of Internal Leakage of Spring Full-Lift Safety Valve Based on Improved Convolutional Neural Network

**DOI:** 10.3390/s25175451

**Published:** 2025-09-03

**Authors:** Shuxun Li, Kang Yuan, Jianjun Hou, Xiaoqi Meng

**Affiliations:** 1 School of Petrochemical Technology, Lanzhou University of Technology, Lanzhou 730050, China; zhds00180081@126.com (S.L.); hou19700018@126.com (J.H.); w18193326164@163.com (X.M.); 2 Machinery Industry Pump Special Valve Engineering Research Center, Lanzhou 730050, China

**Keywords:** spring full-lift safety valve, internal leakage identification, improved CNN, acoustic emission, high-frequency FPGA data acquisition

## Abstract

In modern industry, the spring full-lift safety valve is a key device for safe pressure relief of pressure-bearing systems. Its valve seat sealing surface is easily damaged after long-term use, causing internal leakage, resulting in safety hazards and economic losses. Therefore, it is of great significance to quickly and accurately diagnose its internal leakage state. Among the current methods for identifying fluid machinery faults, model-based methods have difficulties in parameter determination. Although the data-driven convolutional neural network (CNN) has great potential in the field of fault diagnosis, it has problems such as hyperparameter selection relying on experience, insufficient capture of time series and multi-scale features, and lack of research on valve internal leakage type identification. To this end, this study proposes a safety valve internal leakage identification method based on high-frequency FPGA data acquisition and improved CNN. The acoustic emission signals of different internal leakage states are obtained through the high-frequency FPGA acquisition system, and the two-dimensional time–frequency diagram is obtained by short-time Fourier transform and input into the improved model. The model uses the leaky rectified linear unit (LReLU) activation function to enhance nonlinear expression, introduces random pooling to prevent overfitting, optimizes hyperparameters with the help of horned lizard optimization algorithm (HLOA), and integrates the bidirectional gated recurrent unit (BiGRU) and selective kernel attention module (SKAM) to enhance temporal feature extraction and multi-scale feature capture. Experiments show that the average recognition accuracy of the model for the internal leakage state of the safety valve is 99.7%, which is better than the comparison model such as ResNet-18. This method provides an effective solution for the diagnosis of internal leakage of safety valves, and the signal conversion method can be extended to the fault diagnosis of other mechanical equipment. In the future, we will explore the fusion of lightweight networks and multi-source data to improve real-time and robustness.

## 1. Introduction

In the context of the continuous evolution and expansion of the modern industrial system, spring-loaded safety valves are widely used as safety pressure relief devices for various pressure vessels and pressure pipeline networks as key protective devices to ensure safe pressure relief of industrial pressure-bearing systems. However, spring-loaded safety valves are prone to various forms of seat sealing surface damage after long-term opening and closing impact, corrosion, and medium erosion, which may lead to internal leakage, causing huge safety hazards and economic losses to the pressure-bearing equipment system. Rapid and accurate diagnosis and identification of the internal leakage state of spring-loaded safety valves can detect abnormalities in the early stage of internal leakage and provide diagnosis and alarms, which is conducive to the realization of “predictive maintenance” and provides a reference for improving the reliability design of seals, greatly improving the reliability, safety and service life of safety valves and the pressure-bearing equipment systems they protect, and reducing the safety hazards and economic losses caused by medium internal leakage.

The current methods for identifying and diagnosing fluid machinery faults are mainly model-based diagnosis methods and data-driven diagnosis methods [1,2]. However, since many parameters in the fluid machinery model are extremely difficult to determine, such as the flow coefficient, channel geometry, velocity damping coefficient, and flow reaction force, model-based diagnostic methods may not be able to accurately identify whether the valve has internal leakage and the severity of the internal leakage. Data-driven fault identification methods can obtain knowledge from the collected equipment operation data and then apply the learned knowledge to intelligently identify equipment faults, effectively transforming the fault identification problem that previously required professional skills and rich experience into a classification problem [3,4,5]. Many studies have used machine learning methods to identify and classify fluid machinery system faults. Li et al. [6] proposed a hydraulic pump fault diagnosis method that integrates empirical mode decomposition and wavelet kernel extreme learning machine. Araste et al. [7] developed a fluid machinery fault diagnosis method based on electrical signal analysis and support vector machine (SVM). By analyzing the time–frequency characteristics of stator current and voltage signals and combining SVM classifiers, fault mode recognition such as bearing wear and impeller damage can be achieved. Naimi et al. [8] proposed a valve fault detection and diagnosis technology for pressurized water reactor nuclear power plants based on machine learning methods. They used shallow neural networks to detect faults and integrated models such as support vector machines and K-nearest neighbors to identify valve faults. Potočnik et al. [9] constructed a heating valve state classification model based on acoustic signal features and machine learning, extracted Mel frequency cepstral coefficients and linear prediction coding features, and combined random forests with support vector machines to achieve fault identification such as valve jamming and wear. Although machine learning methods have made significant progress in small-sample fault diagnosis, they also face certain challenges. Machine learning-based methods usually rely on manual feature extraction, and the accuracy of feature selection has an important impact on diagnostic performance.

In recent years, deep learning has shown great potential in the field of mechanical fault diagnosis. Inspired by the principles of visual perception, convolutional neural networks are a mainstream deep learning method that have been widely used in image recognition, language and video processing, etc. due to their powerful feature mining and extraction capabilities. Many studies have shown that CNN algorithms can also be used for disease diagnosis, rotating motor fault diagnosis, and other types of mechanical equipment fault detection, identification, or classification tasks. Duan et al. [10] proposed to use convolutional neural networks to solve the problem of fluid machinery bearing fault classification, enhanced feature learning ability through parallel convolution architecture, and combined adaptive cross entropy loss to solve the problem of training with a small number of samples. Yu et al. [11] proposed a CNN-SVM model for airborne fuel pump fault diagnosis, introduced a simulated annealing genetic algorithm to optimize the CNN network structure and parameters, and used SVM to replace the fully connected layer to improve the airborne fuel pump fault classification effect. Jiang et al. [12] proposed an axial piston pump fault diagnosis method based on one-dimensional CNN, decomposed and reconstructed the denoised signal through empirical wavelet transform, and fused the time and frequency domain features into the one-dimensional CNN model to realize axial piston pump fault diagnosis. Dao et al. [13] proposed a turbine fault recognition model that integrates CNN and long short-term memory (LSTM). CNN adaptively extracts and reduces turbine fault features and sends them to the LSTM model for feature learning and training. Zhong et al. [14] proposed a gas turbine fault diagnosis method based on CNN and SVM. Than et al. [15] constructed a bearing fault recognition model using Mamba. Li et al. [16] proposed a lightweight intelligent diagnosis method based on Mamba-ARN to solve the internal leakage problem of natural gas pipeline control valves, combining multiple technologies to improve model performance. Bellacci et al. [17] proposed a wavelet analysis method that captures transient features in signals through joint transformation in the time and frequency domains, thereby realizing the identification and location of short-wavelength faults in railway rail heads. Liang et al. [18] proposed a rolling bearing fault diagnosis method that combines wavelet transform with ResNet. This method performs well in a noisy test environment, and its diagnostic accuracy is significantly better than that of traditional CNN and other improved models. Wavelet transform is more advantageous in processing transient changes in non-stationary signals and transient leakage signals in a strong noise environment. However, the computational complexity of wavelet transform is high, while the computational complexity of short-time Fourier transform is lower than that of wavelet transform, with high computational efficiency. It also realizes localized analysis through a fixed window, making it suitable for capturing steady-state high-frequency features of safety valve internal leakage and more suitable for real-time signal conversion and batch processing of high-frequency FPGA systems. E. Elfatimi et al. [19] classified leaf diseases based on TensorFlow and MobileNet models using public data sets. After optimizing the structure, the average accuracy of training and testing exceeded 97% and 92%, respectively. Bouguezzi, S. et al. [20] proposed the Ad-MobileNet lightweight model, which includes an improved Ad-depth engine and supports multiple activation functions. It has an accuracy of 88.76% on CIFAR-10 and reduces hardware resources by more than 41%. Alajlan, N.N. et al. [21] used TinyML for sleepiness detection and proposed five lightweight models. After quantization and optimization, the accuracy of MobileNet-V2 under DRQ reached 0.9964. Although lightweight models are widely used in edge device fault diagnosis, they use fewer parameters and have fast inference, but their limited ability to extract complex features may lead to the loss of some information when extracting small internal leakage features. Yan Shengqiang et al. [22] proposed the STFT-CBAM-ICNN method, which converts vibration signals into time–frequency graphs and embeds an attention mechanism for rolling bearing fault diagnosis, solving the problem that CNN is difficult to adaptively extract key features. Sun Cuimin et al. [23] proposed a SE-VIT hybrid network, which uses SVM to segment lesions and introduces an attention module. The accuracy rate on the data set reached 97.26%, which facilitates intelligent management. However, the SE module only compresses channel information and may ignore the spatial local characteristics of valve leakage (such as the frequency domain energy concentration related to the groove position). Although the CBAM module combines channel and spatial attention, its computational complexity is large. The selective kernel attention can be dynamically selected through multi-scale convolution kernels, which is more suitable for the fusion of multi-band features of safety valve leakage.

The above studies show that convolutional neural network algorithms are widely used in fault identification and classification in various fields. However, the selection of hyperparameters of classic convolutional neural networks and their existing improved models mainly relies on experience and repeated experiments, lacking the ability to extract time series features and capture multi-scale features, resulting in slow model convergence and low classification and recognition efficiency. In addition, there is currently a lack of cases in which deep learning is used for the identification and diagnosis of different types of internal leakage in valves. To address the above problems, a spring full-lift safety valve internal leakage detection and recognition model based on the Horned Lizard Optimization Algorithm (HLOA) is proposed, which integrates the bidirectional gated recurrent unit (BiGRU) and the selective kernel attention module (SKAM) on the basis of an improved two-dimensional convolutional neural network (CNN). Firstly, the acoustic emission signals of different internal leakage states of the spring full-lift safety valve, collected by high-frequency FPGA, are short-time Fourier transformed to obtain a two-dimensional time–frequency diagram, and then the information features containing the time domain and frequency domain are input into the model. In order to enhance the feature extraction capability and classification performance of each type of internal leakage, the initial learning rate and L2 regularization coefficient of the CNN are optimized by adding the HLOA optimization algorithm to increase the training accuracy and convergence speed of the model; the BiGRU network and SKAM are integrated to enhance the time series feature extraction capability of the signal and improve the model’s ability to capture multi-scale features and to detect and identify different internal leakage states of the spring full-lift safety valve.

The main contributions of this study are as follows:

1. The composite characteristics of “high-frequency transient pulse” and “multi-scale energy distribution” of the acoustic emission signal of the internal leakage of the safety valve: This paper dynamically adapts HLOA and CNN for the first time—optimizes the learning rate and regularization coefficient in CNN by dynamically switching between local fine-tuning and global exploration, so that it can accurately match the characteristic distribution of high-frequency acoustic emission signals—by integrating the BiGRU-SKAM mechanism of “bidirectional time series capture + multi-scale spatial weighting” to extract the time series and spatial features of high-frequency signals of valve leakage. The HLOA-CNN-BiGRU-SKAM collaborative framework is constructed to solve the parameter optimization problem of high-frequency acoustic emission signals and the limitations of feature extraction.
2. According to the time–frequency characteristics of different internal leakage states of safety valves, this paper designs a mixed convolution kernel and applies the combination of random pooling and the LReLU activation function to high-frequency acoustic emission signal processing to enhance the characteristic response of high-frequency acoustic emission signals in weak leakage.
3. The constructed HLOA-CNN-BiGRU-SKAM collaborative framework model is used for the first time in the field of high-frequency acoustic emission signal diagnosis of safety valve internal leakage, which solves the problem of micro-damage detection of the safety valve sealing surface and provides a reusable solution for acoustic emission diagnosis of micro-leakage high-pressure equipment.

## 2. Improved CNN Algorithm Establishment

### 2.1. Horned Lizard Optimization Algorithm

The horned lizard optimization algorithm [24] is a new meta-heuristic algorithm. The horned lizard selects the optimal value through behaviors such as skin darkening or lightening, blood spraying, and movement-escape defense. This work is the first to apply HLOA to the diagnosis of high-frequency acoustic emission signals of safety valve internal leakage. The skin color change strategy can be used to locally fine-tune the hyperparameters in the convolutional neural network and search for better values near the current optimal solution; the use of blood spray defense behavior for global exploration can make the randomly generated candidate solutions jump out of the local optimum and avoid overfitting of the CNN model due to parameter fixation. This behavior is highly adapted to the burst signal characteristics of valve leakage. The HLOA output directly acts on the initialization parameters of the CNN for parameter optimization, laying a stable foundation for subsequent feature extraction.

(1) Darkening or lightening of skin color

The horned lizard can adjust its skin color by adjusting the rate of melanocyte production according to the demand for solar thermal energy. This behavior is simulated in the algorithm through the following Formulas (1) and (2) to update the position of the search agent.

(1) $$\begin{array}{c} {\vec{x}}_{worst}(t)={\vec{x}}_{best}(t)+\frac{1}{2}Light_{1}\sin\left({\vec{x}}_{r_{1}}(t)-{\vec{x}}_{r_{2}}(t)\right)\\ -{\left(-1\right)}^{\sigma }\frac{1}{2}Light_{2}\sin\left({\vec{x}}_{r_{3}}(t)-{\vec{x}}_{r_{4}}(t)\right) \end{array}$$

(2) $$\begin{array}{c} {\vec{x}}_{worst}(t)={\vec{x}}_{best}(t)+\frac{1}{2}Dark_{1}\sin\left({\vec{x}}_{r_{1}}(t)-{\vec{x}}_{r_{2}}(t)\right)\\ -{\left(-1\right)}^{\sigma }\frac{1}{2}Dark_{2}\sin\left({\vec{x}}_{r_{3}}(t)-{\vec{x}}_{r_{4}}(t)\right) \end{array}$$

where ${\vec{x}}_{worst}(t)$ and ${\vec{x}}_{best}(t)$ are the worst and best search agents found, respectively. *r*_1_, *r*_2_, *r*_3_, and *r*_4_ are integer random numbers generated between 1 and the maximum number of search agents, where *r*_1_ ≠ *r*_2_ ≠ *r*_3_ ≠ *r*_4_; and ${\vec{x}}_{r1}(t)$, ${\vec{x}}_{r2}(t)$, ${\vec{x}}_{r3}(t)$, ${\vec{x}}_{r4}(t)$ are the selected *r*_1_, *r*_2_, *r*_3_, and *r*_4_ search agents.

(2) Blood spray defense

Horned lizards defend against enemies by spraying blood from their eyes, and this behavior is simulated as parabolic motion. The following Formula (3) is used to update the position of the search agent, allowing the algorithm to explore more widely in the search space, which helps to jump out of the local optimal solution.

(3) $$\begin{array}{c} \vec{x_{i}}(t+1)=\left[v_{o}\cos\left(\alpha \frac{t}{Max\_iter}\right)+\varepsilon \right]\vec{x_{best}}(t)\\ +\left[v_{o}\sin\left(\alpha -\frac{\alpha t}{Max\_iter}\right)-g+\varepsilon \right]\vec{x_{i}}(t) \end{array}$$

where ${\vec{x}}_{i}(t+1)$ is the new search agent position in the solution search space of the *t* + 1 generation; ${\vec{x}}_{best}(t)$ is the best search agent found; ${\vec{x}}_{i}(t)$ is the current search agent; *Max_iter* represents the maximum number of iterations; *t* is the current iteration; *v*_0_ is set to 1 seg; *α* is set to $\frac{\pi }{2}$; ε is set to 1 × 10^−6^; and *g* is Earth’s gravity, 0.009807 km/s^2^.

(3) Moving to escape

The horned lizard moves randomly and quickly in the environment to avoid predators. The following function Formula (4) is used to simulate this movement to search for agent positions, the best search agent positions, and randomly generated values to increase the global search capability.

(4) $${\vec{x}}_{i}(t+1)={\vec{x}}_{best}(t)+walk\left(\frac{1}{2}-\varepsilon \right){\vec{x}}_{i}(t)$$

where ${\vec{x}}_{i}(t+1)$ is the new search agent position in the solution search space of the *t* + 1th generation, ${\vec{x}}_{best}(t)$ is the best search agent of the *t* generation, *walk* is a random number generated between −1 and 1, ε is a random number generated from the standard Cauchy distribution, and ${\vec{x}}_{i}(t)$ is the current *i*-th search agent in the *t* generation.

(4) α-Melanocyte stimulating hormone (α-MSH) secretion rate

In the algorithm, the α-MSH rate value is determined by calculating the relationship between the fitness value of the current search agent and the best and worst fitness values, and then the position of the search agent is adjusted according to the value. The search agent is represented by Formula (5).

(5) $${\vec{x}}_{i}(t)={\vec{x}}_{best}(t)+\frac{1}{2}\left[{\vec{x}}_{r_{1}}(t)-{\left(-1\right)}^{\sigma }{\vec{x}}_{r_{2}}(t)\right]$$

where ${\vec{x}}_{i}(t)$ is the current search agent; ${\vec{x}}_{best}(t)$ is the best search agent found; *r*_1_ and *r*_2_ are integer random numbers generated between 1 and the maximum number of search agents, where *r*_1_ ≠ *r*_2_; ${\vec{x}}_{r1}(t)$ and ${\vec{x}}_{r2}(t)$ are the selected *r*_1_ and *r*_2_ search agents; and σ is a binary value.

### 2.2. Convolutional Neural Network Improvement Strategies

In view of the time–frequency characteristics of different internal leakage states of the safety valve, a hybrid convolution kernel design is used this time. A 3 × 3 convolution kernel is used to accurately capture the 25 kHz ± 500 Hz narrow-band energy peak of a Φ1 mm single slot to solve the problem that small leakage characteristics are easily submerged by noise; a 5 × 5 kernel is used to cover the 20 kHz~50 kHz wide-band energy area of a Φ2 mm double slot to adapt to the multi-band characteristics of complex leakage. The convolution formula is as follows:

(6) $$x_{i}^{j}=f\left(\sum_{t=1}^{n}W_{i}^{j}*x^{j-1}+\alpha_{i}^{j}\right)$$

where $x_{i}^{j}$ represents the *i*-th feature of the output value of the *j*-th layer; $W_{i}^{j}$ represents the weight matrix of the *i*-th convolution kernel of the *j*-th layer; $x^{j-1}$ represents the output of the *j*-1-th layer; $\alpha_{i}^{j}$ is the bias term corresponding to the *j*-th layer; and $f\left(\cdot \right)$ is the activation function.

The output of the ReLU neuron is always zero when the input is negative, and the ReLU neuron always stops learning. Therefore, this paper introduces the Leaky ReLU activation function [25], which can solve these problems by yielding a small positive value when the input is negative, helping the neuron to continue working and strengthening the characteristic response to weak leakage signals. The function expression of LReLU is as follows:

(7) $$f\left(x\right)=\left\{\begin{array}{l} x,\;\;\;\;if\;x>0\\ \alpha x,\;\;if\;x\leq 0 \end{array}\right.$$

where *x* is the input value; and *α* is a small fixed slope (usually set to 0.01 or 0.2).

The pooling layer performs data dimensionality reduction on the feature values extracted by the convolution layer. This paper uses random pooling to fuse the contributions of all high-frequency energy points in the time–frequency graph to avoid missing the weak energy at the intersection of the two slots and to solve the problem that the maximum pooling loses small key information and the average pooling weakens the contribution of some important feature elements, and the randomness of the pooling helps prevent the model from overfitting [25]. The formula is as follows:

(8) $$p_{i,j}=a_{i,j}/\left(\sum_{\left(i,j\right)\in R_{S}}a_{i,j}\right)$$

where $p_{i,j}$ is the probability of element $a_{i,j}$, and *R_S_* is the pooling area.

The fully connected layer combines and maps the pooled features into a one-dimensional feature vector of fixed length and inputs them into the classifier for classification after nonlinear activation of the activation function. The expression of the fully connected layer is as follows:

(9) $$g^{l}=f\left(W^{l}g^{l-1}+\varepsilon^{l}\right)$$

where *g^l−^*^1^ is the output layer of the previous network; *W^l^* is the weight; *g^l^* is the output layer of the fully connected layer; $f\left(\cdot \right)$ is the activation function; and $\varepsilon^{l}$ is the bias parameter.

**Figure 1:**
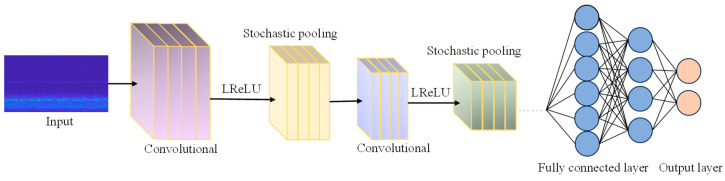
Improved convolutional neural network structure diagram.

The structure diagram of the improved hybrid convolution kernel neural network is shown in Figure 1:

### 2.3. Bidirectional Gated Recurrent Unit

**Figure 2:**
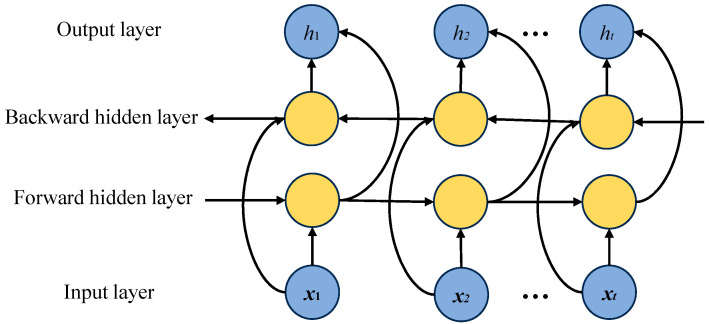
BiGRU structure diagram.

In order to solve the problem of gradient vanishing and gradient exploding when processing long sequence data, this paper introduces the BiGRU bidirectional gated recurrent unit, which consists of an update gate, a reset gate, and a bidirectional structure [26,27,28]. The bidirectional structure forward GRU extracts the temporal dependency from 0 to t, and the reverse GRU captures the feature association from t to 0 and jointly outputs the complete temporal feature vector of the acoustic emission signal to improve the convergence speed and prediction ability of the model. The BiGRU structure is shown in Figure 2.

The mathematical expression of the BiGRU model is as follows:

(10) $$\vec{h_{t}}=G(x_{t},\vec{h_{t-1}})$$

(11) $$\overset{\leftarrow }{h_{t}}=G(x_{t},\overset{\leftarrow }{h_{t-1}})$$

(12) $$h_{t}=f(\vec{Wh_{t}}+\overset{\leftarrow }{Wh_{t}}+b_{t})$$

where *G*( ) is the combination of the update gate, reset gate, and candidate state of each unidirectional GRU; $\vec{h_{t}}$, $\vec{h_{t-1}}$ are the forward hidden layer states at time *t* and *t* − 1; $\overset{\leftarrow }{h_{t}}$, $\overset{\leftarrow }{h_{t-1}}$ are the backward hidden layer states at time *t* and *t* − 1; *f*( ) is the activation function; $\vec{W}$, $\overset{\leftarrow }{W}$ are the forward and backward hidden layer weights at time *t*; and $b_{t}$ is the hidden layer bias at time *t*.

### 2.4. Selective Kernel Attention Module

**Figure 3:**
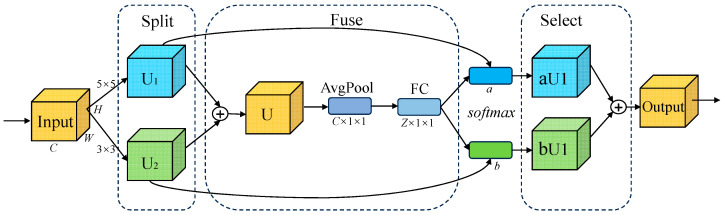
SK attention module structure diagram.

In order to extract spatial features of the high-frequency signal of safety valve leakage, we introduced the SK attention module, which can selectively fuse convolution kernels of different scales, allowing the network to adaptively select appropriate convolution kernels according to the input features to capture multi-scale features, thereby improving the model feature extraction performance [29,30]. The SK attention module consists of three parts: split, fusion, and selection. Its structure is shown in Figure 3.

The Split part uses 3 × 3 and 5 × 5 convolution kernels to perform convolution operations on the input feature map to obtain two feature maps of different scales, U1 and U2; the Fuse part sums the two feature maps of different scales, U1 and U2, to generate channel statistics U, and then obtains the channel descriptor S through global average pooling. Then, a fully connected layer (FC) is used to reduce the dimension of the channel descriptor to complete the extraction of the information channel dimension. The expression of Fuse is as follows:

(13) $$U=U_{1}+U_{2}$$

(14) $$S_{C}=\frac{1}{H\times W}\sum_{i=1}^{H}\sum_{j=1}^{W}U_{c}\left(i,j\right)$$

(15) $$Ζ=\delta \left(W_{1}s\right)$$

where *c* represents the channel index; *H* and *W* are the height and width of the feature map, respectively; *W*_1_ is the weight matrix of the fully connected layer; *δ* is the ReLU activation function; and *Z* is the compact feature descriptor.

The Select part normalizes through the SoftMax function, calculates the weight score corresponding to each channel, and multiplies the weight with the corresponding feature map and adds them to fuse the information to obtain the final output image.

The expression of Select is as follows:

(16) $$a_{c}=\frac{e^{A_{c}Z}}{e^{A_{c}Z}+e^{B_{c}Z}},b_{c}=\frac{e^{B_{c}Z}}{e^{A_{c}Z}+e^{B_{c}Z}}$$

(17) $$V_{c}=a_{c}\cdot U_{1}+b_{c}\cdot U_{2},a_{c}+b_{c}=1$$

where *a* and *b* represent the weight scores of the soft attention vectors for U_1_ and U_2_, respectively; and *V* represents the final feature map of the output.

### 2.5. Improved 2D CNN Model Framework

**Figure 4:**
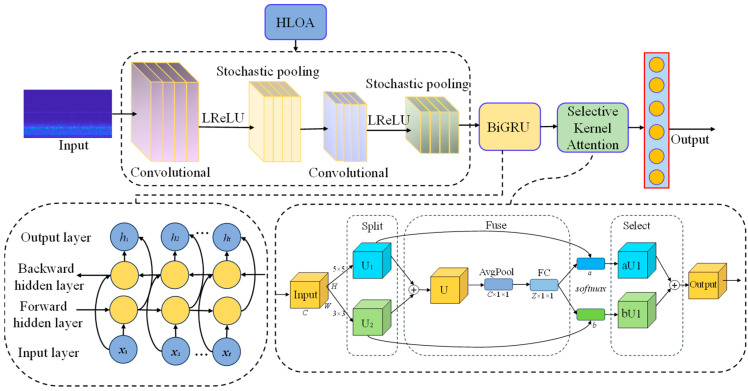
Improved two-dimensional CNN model framework.

First, the LReLU activation function is used to replace the ReLU activation function in the traditional convolutional neural network to improve the nonlinear expression ability. Since random pooling integrates all feature elements and can effectively prevent model overfitting, random pooling is used to replace maximum pooling and average pooling. The HLOA optimization algorithm is used to adaptively learn the initial learning rate and L2 regularization coefficient of the parameters in the convolutional neural network, and the optimal initial learning rate and L2 regularization coefficient are found to improve the training accuracy of the model. The BiGRU network is embedded behind the convolution layer and the pooling layer. The long-term and short-term dependencies in the sequence data can be effectively captured through the bidirectional recurrent unit, and features can be further extracted from the two-dimensional matrix in the time series direction. SKAM is embedded between the BiGRU and the fully connected layer, so that the network can adaptively select the appropriate convolution kernel according to the input features to capture multi-scale features, thereby improving the model performance. Finally, each state is finally classified through the fully connected layer and the SoftMax classifier. The improved two-dimensional CNN model framework is shown in Figure 4.

### 2.6. The Overall Process of Identifying Internal Leakage of Spring Full-Lift Safety Valve

**Figure 5:**
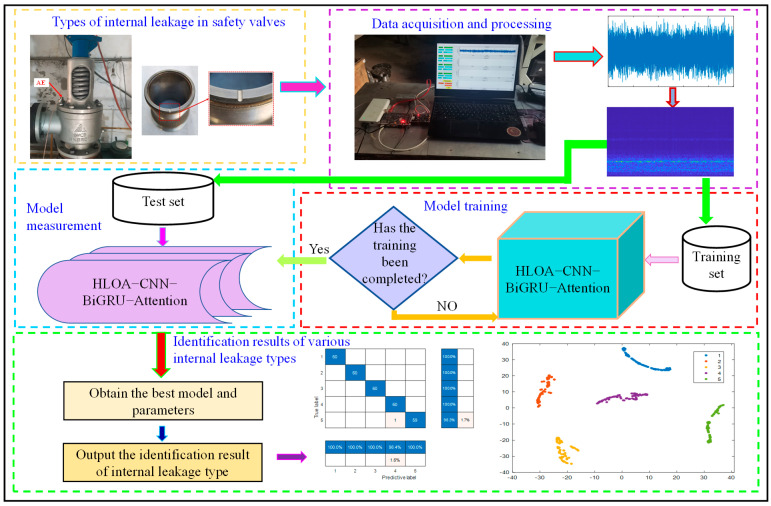
Overall flow chart of internal leakage status identification of spring full-lift safety valve.

The overall process of spring full-lift safety valve internal leakage identification is shown in Figure 5. First, the collected acoustic emission signals of different internal leakage states are processed into two-dimensional time–frequency feature images by short-time Fourier transform. The processed feature images are divided into 70% as training sets and 30% as test sets. The two-dimensional time–frequency feature images of spring full-lift safety valves without internal leakage and various types of internal leakage are packaged and labeled, and stored in different folders. Then, the training set of two-dimensional time–frequency feature maps of various types of internal leakage of spring full-lift safety valves is input into the improved convolutional network for model training. After each training, the recognition accuracy of no internal leakage and various types of internal leakage is evaluated. If the expected accuracy is not achieved, the model training is continued; otherwise, the training is stopped, and the trained model is saved. Finally, the test set of two-dimensional time–frequency feature maps of spring full-lift safety valves without internal leakage and various types of internal leakage is input into the trained model to verify its performance, and the final prediction results of acoustic emission signals of spring full-lift safety valves without internal leakage and various types of internal leakage are output to confirm the effectiveness of the model.

## 3. Acoustic Emission Test Data Collection and Preprocessing of the Healthy and Internal Leakage States of the Safety Valve

### 3.1. Fault Presetting of Safety Valves in Different Internal Leakage States

**Figure 6:**
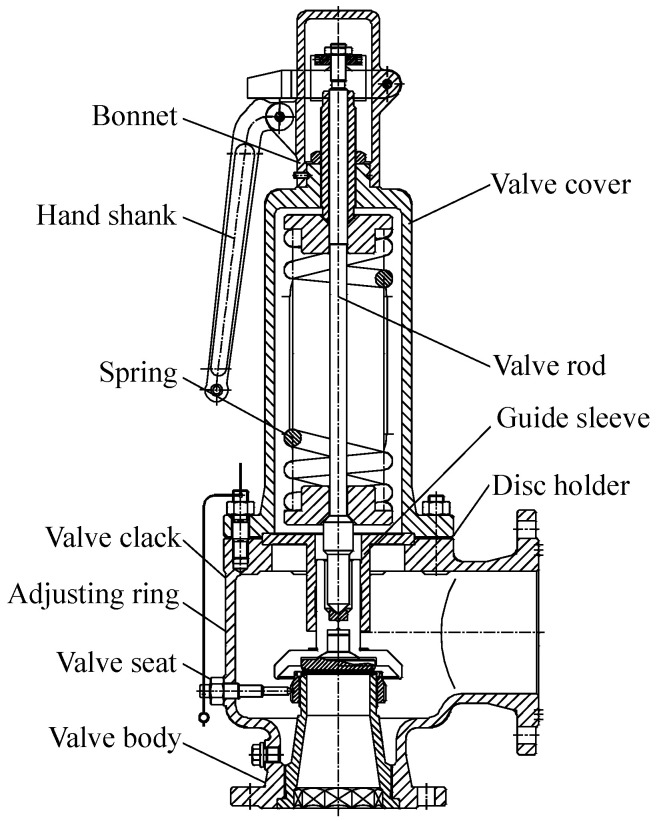
Structure diagram of spring full-lift safety valve.

This experiment takes the PN25 DN100 spring full-lift safety valve as the research object, as shown in Figure 6. The valve is mainly composed of the valve body, valve cover, valve stem, valve seat, valve disc, spring, and other components.

**Figure 7:**
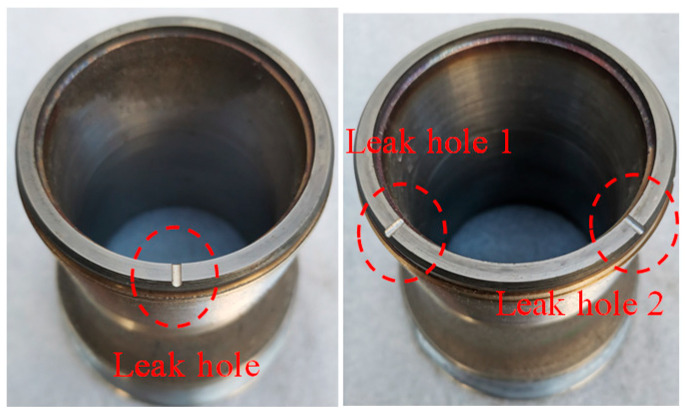
Valve seat leakage failure picture.

By referring to the typical scale and leakage form of common valve seat wear in industrial sites, we found that there are single tiny defects to multi-position complex defects. Therefore, we machined single-semicircular-groove valve seats with certain equivalent diameters of Φ1 mm and Φ2 mm on the sealing surface of the valve seat to simulate progressive damage in a single position, and machined double-semicircular-groove valve seats with certain equivalent diameters of Φ1 mm and Φ2 mm to simulate multi-source damage in multiple positions. This valve seat leakage form conforms to the typical failure form of safety valves subjected to long-term medium erosion and can also reflect the status of different internal leakages in actual operation. The pictures of the actual single-semicircular-groove valve seat and the double-semicircular-groove valve seat are shown in Figure 7.

Therefore, there are five types of health status and leakage status of the spring full-lift safety valve, as shown in Table 1.

### 3.2. Signal Acquisition Experiment Under Different Internal Leakage Forms of Safety Valve

**Figure 8:**
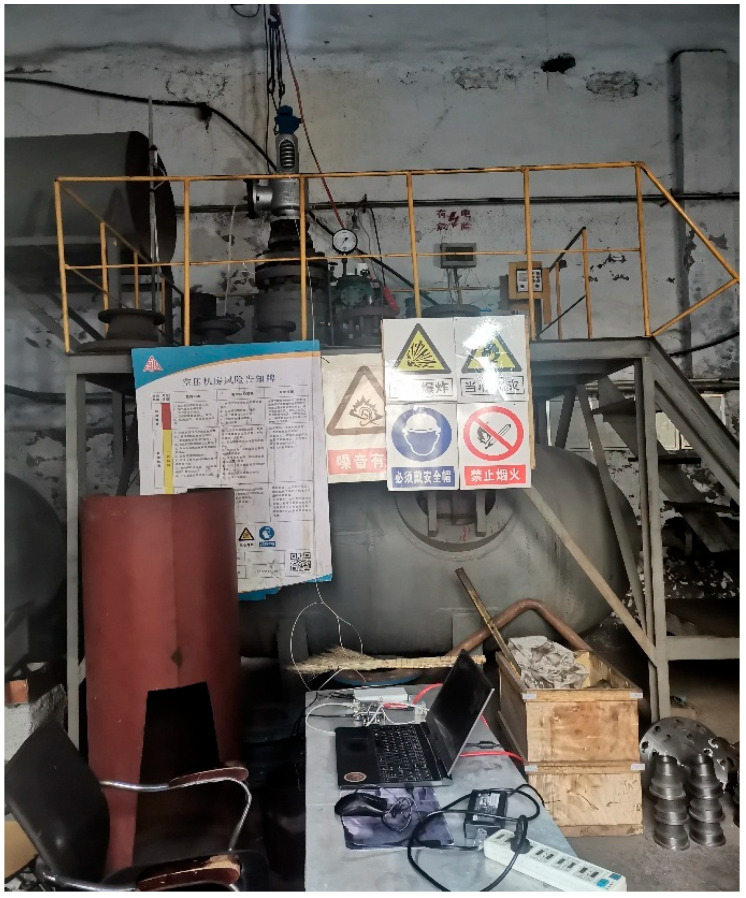
Picture of the actual internal leakage test experiment of the spring full-lift safety valve.

**Figure 9:**
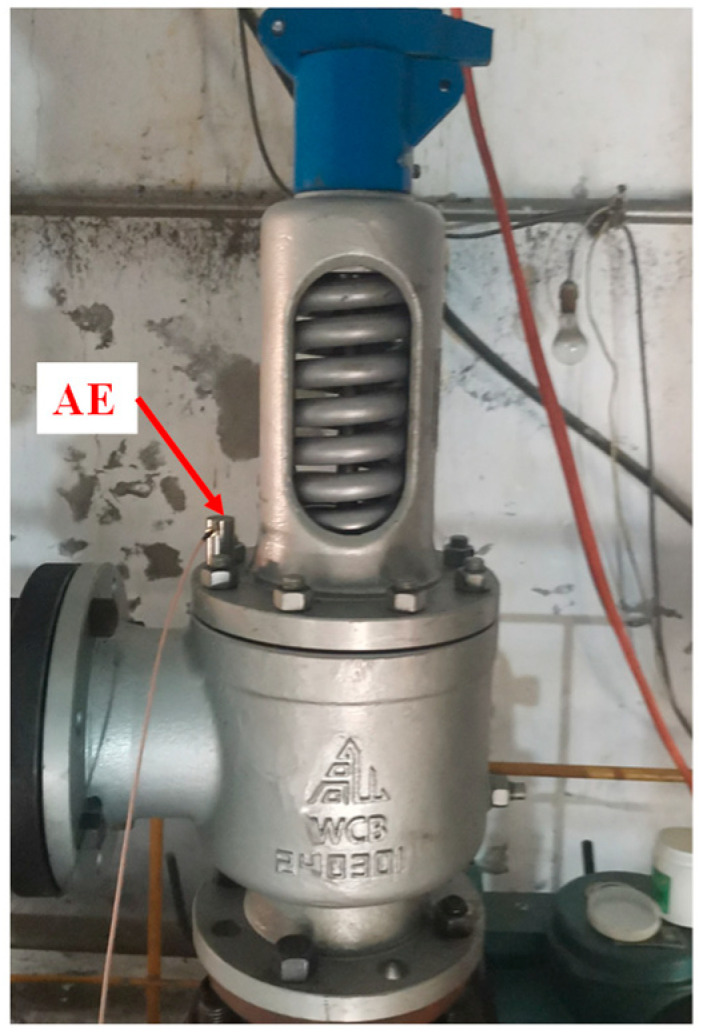
Acoustic emission sensor arrangement measurement point diagram.

The internal leakage test experimental system of the spring full-lift safety valve is shown in Figure 8, and the acoustic emission sensor measurement point arrangement is shown in Figure 9. The internal leakage test experimental system includes a pressure storage tank, a compressor, a pressure gauge, a spring full-lift safety valve to be tested, an acoustic emission sensor, and a high-frequency FPGA data acquisition system. The compressor provides a pressure gas source stored in the pressure storage tank. When the spring full-lift safety valve leaks, the compressed gas flows to the safety valve through the pressure storage tank, and flows to the downstream of the system through the internal leakage groove preset in the safety valve seat to generate an acoustic emission signal. The spring full-lift safety valves equipped with valve seats of different internal leakage types are installed on the test bench in turn, and the acoustic emission sensor is arranged on the upper-end face of the flange of the spring full-lift safety valve. The acoustic emission signals of different internal leakage types of the spring full-lift safety valve are collected using the high-frequency FPGA acoustic emission data acquisition system.

**Figure 10:**
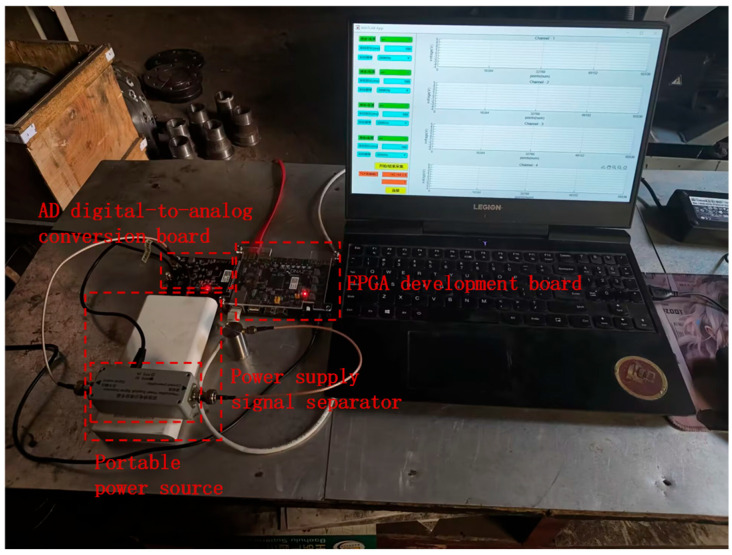
Picture of the actual high-frequency FPGA data acquisition system.

The picture of the actual high-frequency data acquisition system for the acoustic emission signal of the internal leakage of the spring full-lift safety valve is shown in Figure 10. The data acquisition system consists of a preamplifier integrated acoustic emission sensor (sampling frequency dimension 15 kHz~70 kHz), a power supply signal separator (Guangzhou Qingcheng Acoustic Emission Research Co., Ltd., Guangzhou, China), an AD9238 digital-to-analog conversion board, an FPGA development board (Shanghai Xinyi Electronic Technology Co., Ltd., Shanghai, China), a host computer, a mobile power supply, etc.

### 3.3. Time Domain and Short-Time Fourier Time–Frequency Transform Analysis of Acoustic Emission Signals of Different Internal Leakage Types

**Figure 11:**
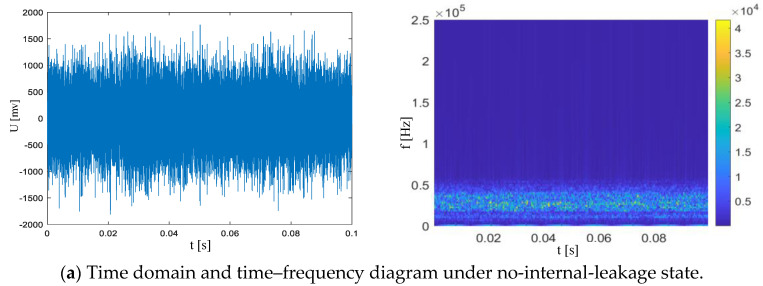

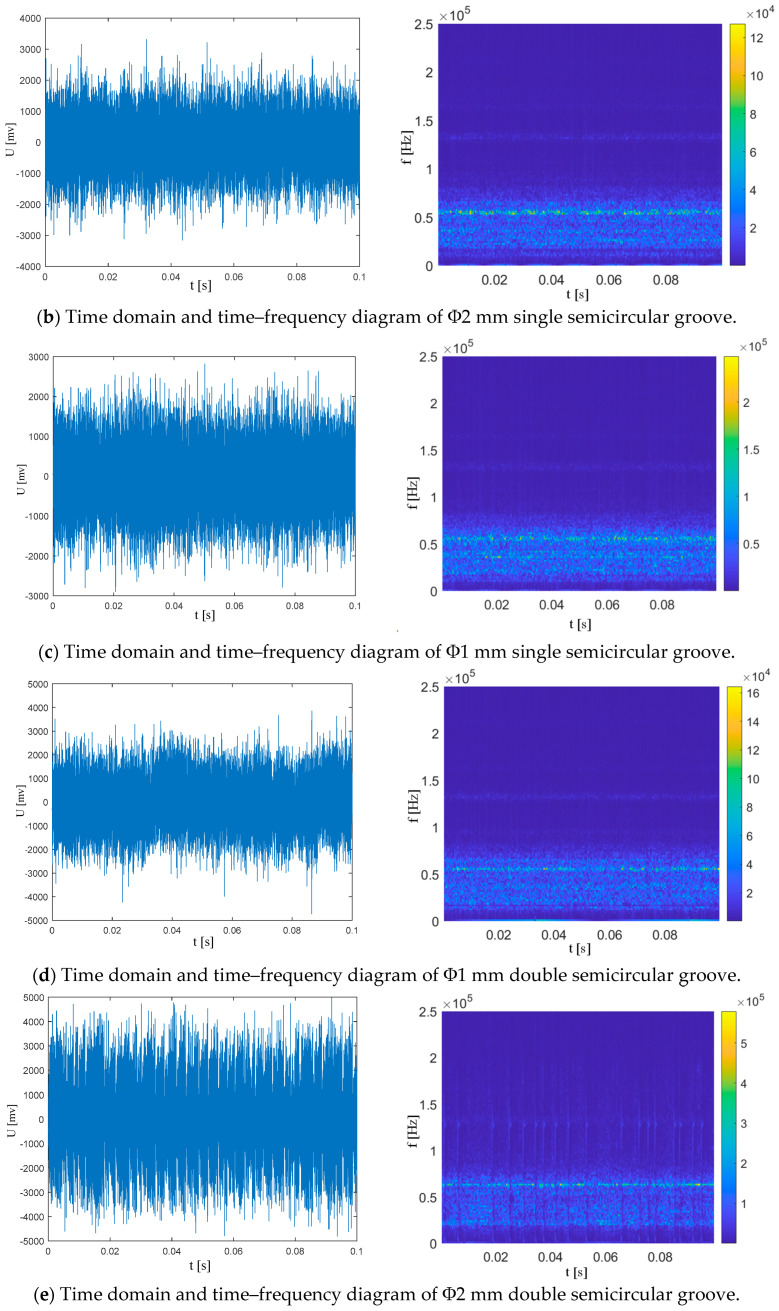
Time domain waveforms of acoustic emission signals under five working conditions (**left column**) and their short-time Fourier transform time–frequency diagrams (**right column**): (**a**) The time domain waveform in the no-internal-leakage state shows a low-amplitude stable feature, and the energy of the time–frequency diagram is mainly concentrated in the low-frequency band below 20 kHz; (**b**) The time domain waveform of the Φ2 mm single-semicircular-groove leakage contains high-frequency-pulse amplitude (about 2 × 10^4^), and the energy of the 20 kHz~40 kHz frequency band of the time–frequency diagram is significantly enhanced, showing a continuous distribution feature; (**c**) The time domain pulse amplitude (about 1.5 × 10^4^) of the Φ1 mm single-semicircular-groove leakage is lower than that of the Φ2 mm single groove, and the high-frequency energy of the time–frequency diagram is concentrated in the 25 kHz ± 5 kHz frequency band; (**d**) The time domain pulse interval of the Φ1 mm double-semicircular-groove leakage is irregular, and the time–frequency diagram forms a “cross-shaped” energy distribution due to the 90° angle between the double grooves (there are two energy peaks at 25 kHz and 35 kHz); (**e**) The Φ2 mm double-semicircular-groove leakage has the largest amplitude in the time domain (about 2.5 × 10^4^), and the time–frequency diagram covers the full frequency band of 20 kHz~50 kHz, and the intensity is significantly higher than the other internal leakage states. The time–frequency diagram uses a Hanning window (length 0.1 s, overlap 75%), and the color bar represents the normalized energy density (dB).

Since the acoustic emission signals generated by the internal leakage of the safety valve are mainly distributed in the ultrasonic frequency band of 20 kHz~50 kHz, the signal duration is short (a single turbulent pulse is about 0.01~0.05 s). In order to fully capture the entire process of the acoustic emission signal and its attenuation, a window length of 0.1 s is selected for the collected acoustic emission time domain signals of the spring full-lift safety valve without internal leakage and the acoustic emission time domain signals of various types of internal leakage, and a short-time Fourier transform is performed to obtain a two-dimensional time–frequency diagram, as shown in Figure 11. The STFT analysis in Figure 11 uses a Hanning window (2048 points, 50% overlap) and achieves a frequency resolution of 34.18 Hz at a sampling frequency of 70 kHz, accurately capturing the differences in time–frequency characteristics under different leakage states. It can be seen from the time–frequency spectrum that most of the energy of the acoustic emission signal without internal leakage is concentrated in the relatively low-frequency band area, but the acoustic emission signals of different types of internal leakage are concentrated in the ultrasonic frequency band of 20 kHz~50 kHz, and there is also a large amount of energy distribution below the 20 kHz ultrasonic frequency band. The energy distributions of the no-internal-leakage frequency band of the spring full-lift safety valve and the different internal leakage types overlap seriously, and it is impossible to judge the internal leakage state of the spring full-lift safety valve from the two-dimensional time–frequency spectrum. Therefore, a recognition model based on deep learning CNN training is proposed to accurately and effectively identify the different internal leakage states of the spring full-lift safety valve.

## 4. Identification of Internal Leakage Status of Spring Full-Lift Safety Valve

### 4.1. Establishment of Data Set and Label for Identification of Internal Leakage Status of Spring Full-Lift Safety Valve

The training set and test set of this study were divided by stratified random sampling combined with time batch separation. The experimental data were collected in three batches (each batch was separated by 24 h to control the consistency of interference such as ambient temperature and pressure): for five internal leakage conditions, 200 groups of samples for each internal leakage condition—about 140 groups of experimental data collected from batches 1 and 2 were randomly selected as training sets (70%), and about 60 groups of experimental data collected from batches 3 and the first two batches were randomly selected as test sets (30%). This data set division method can ensure that the proportion of each condition in the training set and the test set is strictly consistent (both 20%) and consistent in time sequence independence, avoiding evaluation bias caused by a too high or too low proportion of a certain type of sample in the test set, and is closer to the actual application scenario, which can verify the model’s recognition ability for newly collected data.

The data set divided by stratified random sampling combined with time batch separation is transformed into a two-dimensional time–frequency diagram through short-time Fourier transform. The total database sample set includes 200 two-dimensional time–frequency diagrams of acoustic emission without internal leakage and 200 two-dimensional time–frequency diagrams of acoustic emission of four different types of internal leakage, totaling 1000 two-dimensional time–frequency diagrams of acoustic emission. The total data set is divided into 70% for the training set and 30% for the test set, and the training set database is 700 two-dimensional time–frequency diagrams of acoustic emission, and the test set is 300 two-dimensional time–frequency diagrams of acoustic emission. The number of divisions and label settings of the training set and test set under different forms of internal leakage is shown in Table 2 below.

### 4.2. Analysis on the Accuracy of Internal Leakage Identification of Spring Full-Lift Safety Valve

**Figure 12:**
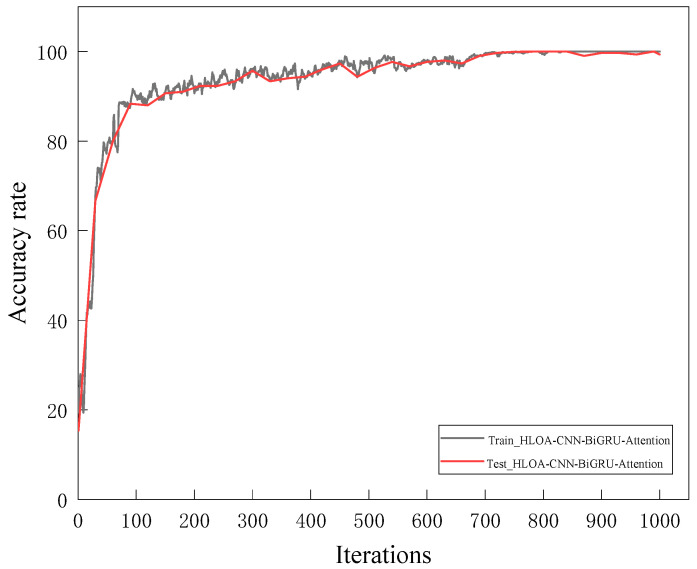
HLOA-CNN-BiGRU-Attention model accuracy curve.

**Figure 13:**
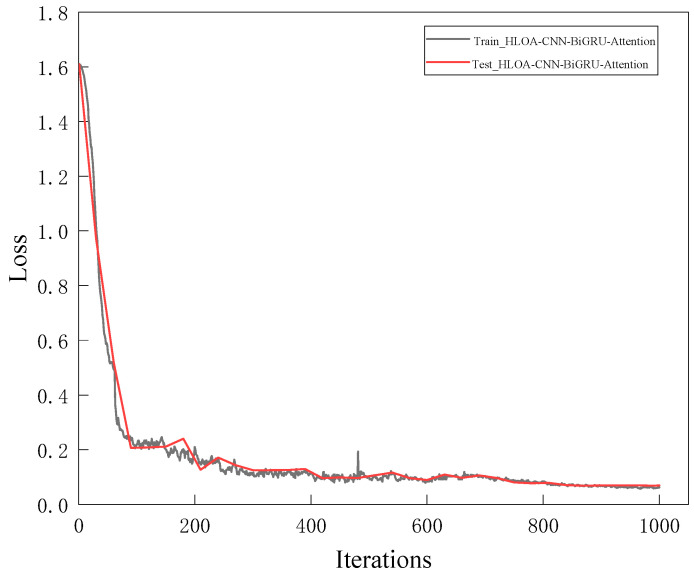
HLOA-CNN-BiGRU-Attention model loss rate curve.

The training set of two-dimensional time–frequency feature maps of the spring full-lift safety valve without internal leakage and various internal leakage types is input into the HLOA-CNN-BiGRU-Attention model for model training, and the training results are used to determine whether the training accuracy requirements are met. If they are met, the trained model is saved; then, the test set is input into the saved model for testing. Figure 12 shows the training accuracy and test accuracy of the HLOA-CNN-BiGRU-Attention model; Figure 13 shows the training loss and test loss of the HLOA-CNN-BiGRU-Attention model. It can be seen from the figures that the HLOA-CNN-BiGRU-Attention model performs evenly on the training set and the test set, and begins to converge after the number of iterations is greater than 100. When the number of iterations reaches 700, the accuracy curve and the loss curve tend to be stable, reaching a higher accuracy and a lower loss value. The training accuracy quickly converges to a higher level, close to 100%; so, the improved model has a higher recognition ability.

This experiment uses an NVIDIA RTX 3090 (24 GB VRAM), Intel i9-12900K CPU, 64 GB RAM server, and NVIDIA Jetson AGX Xavier (384-core Volta GPU, 16 GB RAM) inference edge device. The 1000 sets of time–frequency graphs are divided into batch size = 32, and eight GPUs are used for parallel computing to accelerate model convergence. The total training time is about 4.2 h, which is 38.2% shorter than the 6.8 h of traditional CNN (ResNet-18). From the accuracy curve of the HLOA-CNN-BiGRU-Attention model in Figure 13, it can be seen that the training accuracy reached 99% in the 480th round. The inference time of the server-side RTX 3090 to process a single time–frequency graph (224 × 224 pixels) is 12.7 ms, which meets the millisecond-level diagnosis requirements; the inference time of the edge-side Jetson AGX Xavier is 21.3 ms, which is lower than the real-time threshold (50 ms) for safety valve diagnosis on industrial sites and can achieve about 47.2 continuous detections per second (FPS = 47.2).

**Figure 14:**
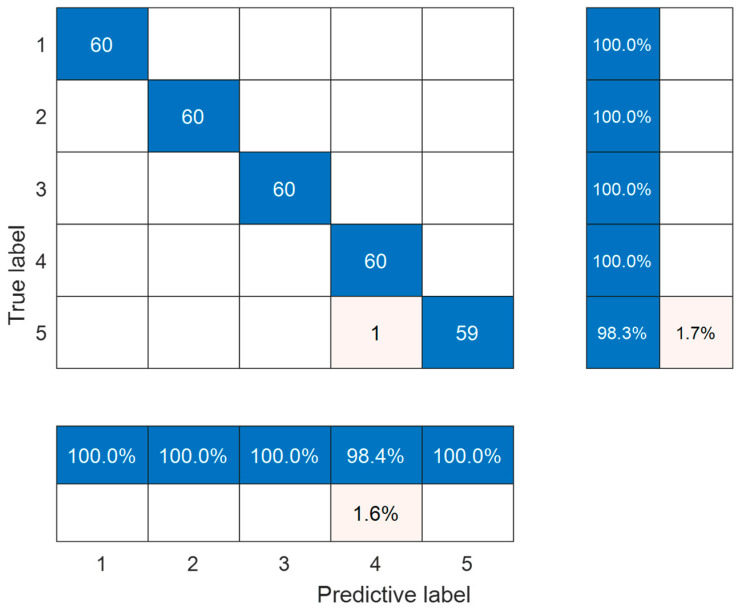
HLOA-CNN-BiGRU-Attention model confusion matrix.

**Figure 15:**
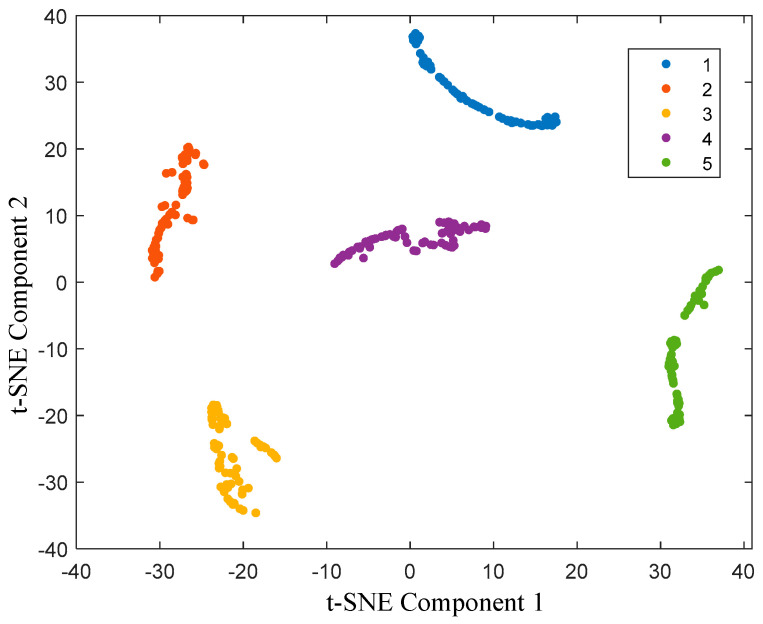
HLOA-CNN-BiGRU-Attention model t-SNE visualization.

The confusion matrix of the HLOA-CNN-BiGRU-Attention model on the test set samples is shown in Figure 14. From the confusion matrix, it can be seen that each internal leakage condition contains 60 samples, a total of five different internal leakage conditions, and a total of 300 test set samples; the HLOA-CNN-BiGRU-Attention model can classify all types of samples from labels 1 to 4, and one sample in label 5 (Φ2 mm double semicircular groove) is misclassified as label 4. Table 3 shows the precision, recall, and F1 score after the calculation of the confusion matrix. In the table, TP is the number of samples correctly identified in the i-th class, FP is the number of samples of other classes misclassified as the *i*-th class, and FN is the number of samples of the i-th class misclassified as other classes. Through the analysis of Figure 15 and Table 3, it can be seen that the proposed HLOA-CNN-BiGRU-Attention model can achieve high-precision intelligent recognition of spring full-open safety valves without internal leakage and various types of internal leakage.

In order to further illustrate the classification effect of the spring full-lift safety valve state recognition model, the t-SNE nonlinear dimensionality reduction algorithm is introduced to visualize the sample features of the final output layer, as shown in Figure 15. It can be seen from Figure 15 that the categories of the output layer are compactly distributed, with clear boundaries and almost no overlap. The HLOA-CNN-BiGRU-Attention model successfully separates the five different spring full-lift safety valve internal leakage categories, and it has advantages in dimensionality reduction and maintaining data structure integrity. The HLOA-CNN-BiGRU-Attention model performs well in category visualization and separation effects.

In this study, the cluster distribution of the t-SNE graph essentially reflects the characteristic differences of the acoustic emission signals under different leakage conditions. In the experiment, as the aperture increases from 1 mm to 2 mm, the turbulence intensity of the leaking airflow increases, resulting in a decrease in the energy proportion of the high-frequency band and an increase in the energy amplitude of the low-frequency band, which makes the two form independent clusters with clear boundaries in the t-SNE graph. From the t-SNE graph, it can be seen that the clusters of the 1 mm aperture samples are relatively concentrated in the t-SNE graph and have no disconnection, such as the clusters represented by the red and purple points in the figure, while the clusters of the 2 mm aperture samples are relatively dispersed in the t-SNE graph and have obvious disconnection compared with the clusters of the 1 mm aperture, such as the clusters represented by the yellow and green points in the figure. This distribution pattern is consistent with the trend of acoustic feature changes caused by the increase in aperture; that is, the feature evolution brought about by the increase in diameter is manifested as the gradual migration of sample points along a specific direction after t-SNE dimensionality reduction, reflecting the model’s sensitive capture of the change in the continuous parameter of aperture. For intermediate apertures that do not appear in the training set, the recognition result of the model depends on the similarity between its features and the training samples. Since the energy distribution and frequency components of the acoustic emission signal generated by the leakage change regularly when the aperture increases from 1 mm to 2 mm, the model will make judgments based on this continuous feature evolution: if the intermediate aperture signal features are closer to the 1 mm sample (such as the high-frequency component of 1.3 mm is high and small), it tends to be identified as 1 mm; if the features are closer to the 2 mm sample (such as the low-frequency energy of 1.5 mm is more significant), it tends to be identified as 2 mm. In subsequent research, we will add more experimental data on intermediate apertures to further verify this feature evolution law to enhance the model’s explanatory power for continuous parameter changes.

### 4.3. Ablation Experiment Analysis of HLOA-CNN-BiGRU-Attention Model

**Figure 16:**
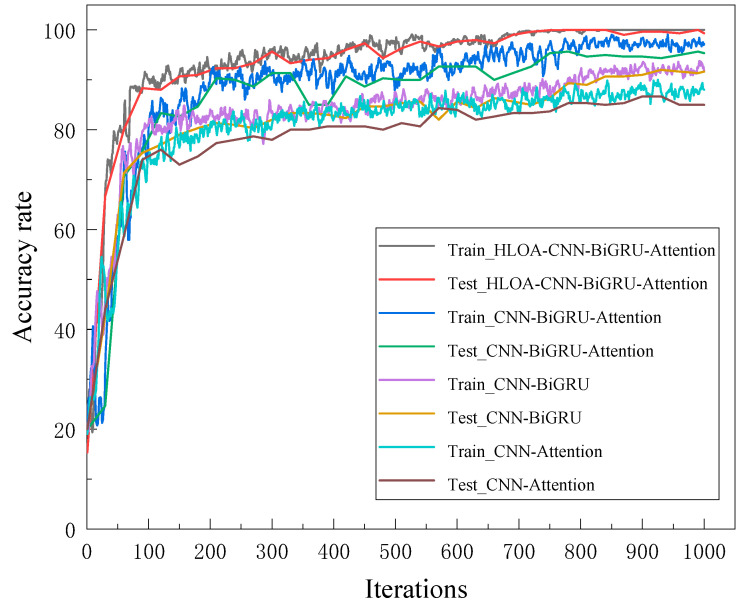
Ablation experiment training and test accuracy curve.

**Figure 17:**
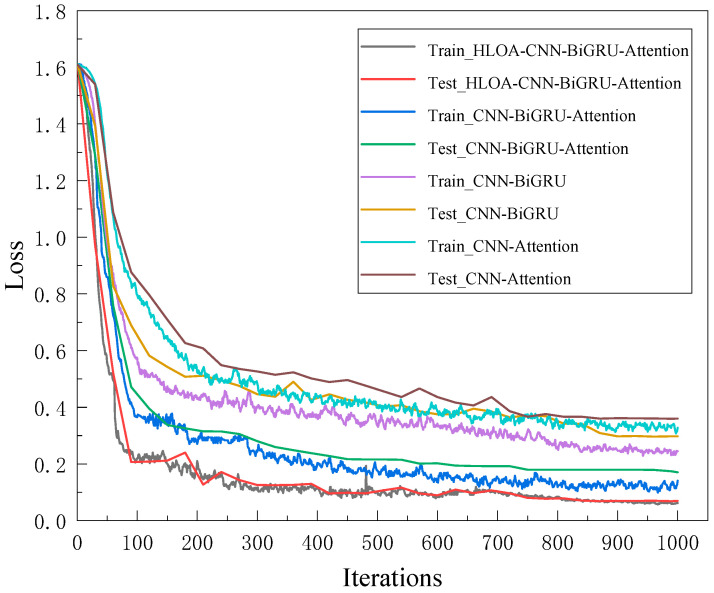
Ablation experiment training and test loss rate curve.

In order to further verify the necessity of each module in the proposed HLOA-CNN-BiGRU-Attention model, an ablation experiment was conducted. To ensure the fairness of each experiment, the same training rounds, learning rate, learning rate decay factor, and batch size were maintained in each ablation experiment. Figure 16 and Figure 17 show the accuracy and loss values in the ablation experiment. It can be seen from Figure 16 that during the entire training process, the use of the CNN-Attention module has the greatest impact on the model prediction accuracy, followed by the use of the CNN-BiGRU module, while the use of the CNN-BiGRU-Attention module has the least impact on the model prediction accuracy. Figure 17 shows that the loss obtained by training with the CNN-BiGRU-Attention module is significantly higher than that with the proposed HLOA-CNN-BiGRU-Attention model.

**Figure 18:**
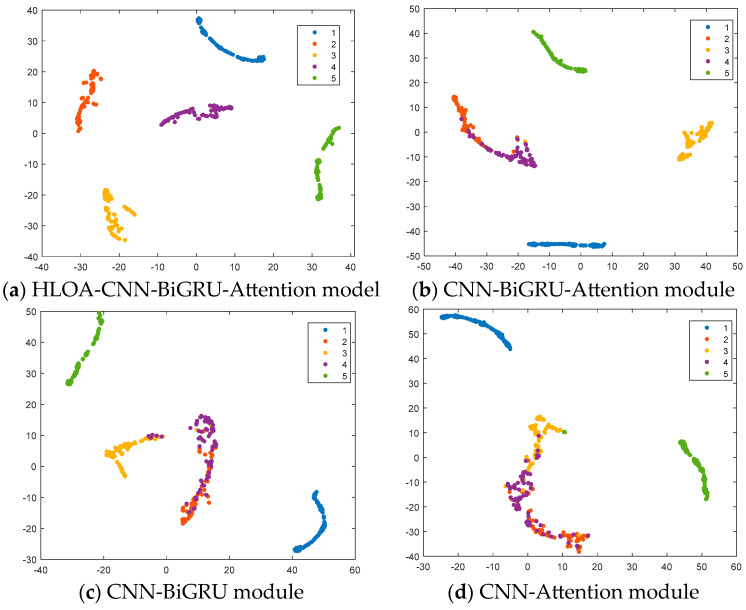
t-SNE diagram of ablation experiment.

Figure 18 shows the feature distribution results of the ablation experiment. The t-SNE results in the figure show the feature distribution of different types of internal leakage of the spring full-lift safety valve. In the ablation experiment, the HLOA-CNN-BiGRU-Attention model performed best, successfully separating the five categories 1 to 5 clearly. The distribution of each category was compact, the boundaries were obvious, and there was almost no overlap, reflecting its advantages in dimensionality reduction and maintaining the integrity of the data structure. In contrast, the CNN-BiGRU-Attention module performed worse in the t-SNE map, with blurred boundaries between categories 2 and 4 and a small amount of overlap. There was significant overlap between categories 2 and 4 in the t-SNE map of the CNN-BiGRU module; there was a significant aliasing phenomenon in categories 2, 3, and 4 in the t-SNE map of the CNN-Attention module; and its classification ability was still poor compared with the HLOA-CNN-BiGRU-Attention model. Overall, after removing each module from the HLOA-CNN-BiGRU-Attention model, the performance of the category visualization and separation effect declined.

### 4.4. Analysis of Algorithm Superiority

**Figure 19:**
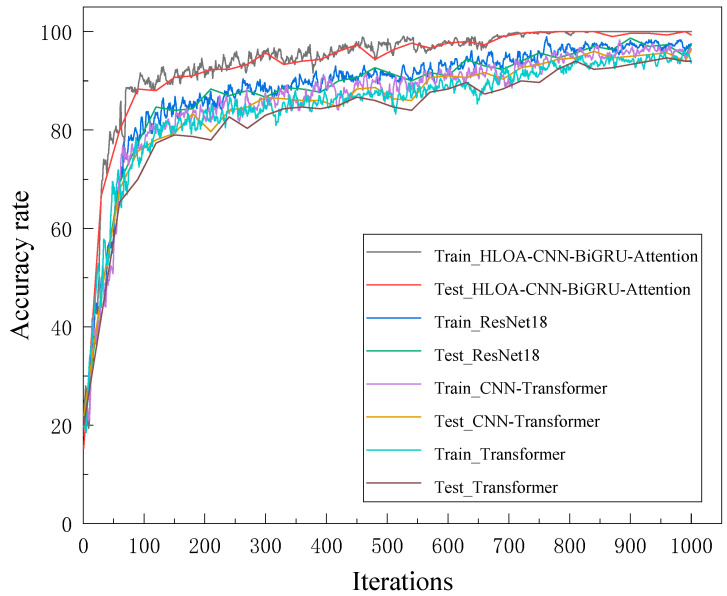
Training and testing accuracy curves of various algorithms.

**Figure 20:**
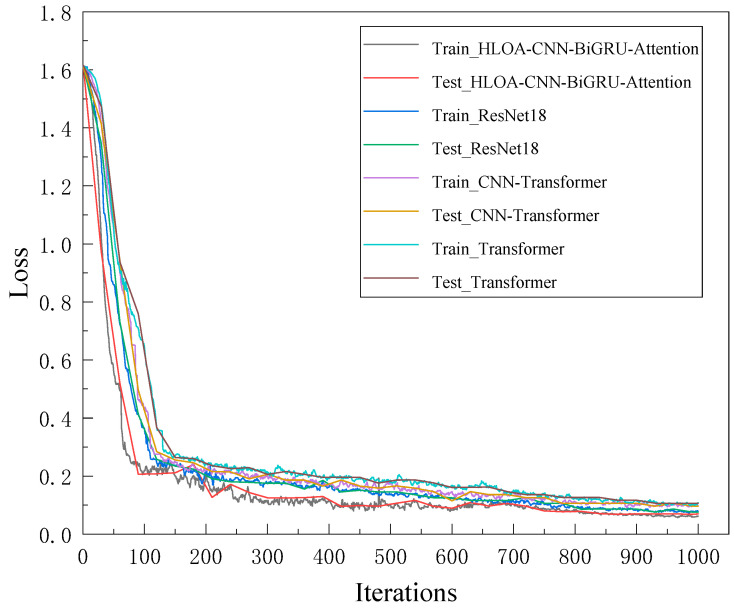
Training and testing loss rate curves of various algorithms.

In order to verify the effectiveness of the proposed model, three mainstream methods, Transformer, ResNet-18, and CNN-Transformer, are used for comparison. Figure 19 and Figure 20 show the training accuracy, test accuracy, training loss, and test loss of the four methods. As can be seen from the figure, the HLOA-CNN-BiGRU-Attention model has the most balanced performance on the training set and the test set, achieving a higher accuracy and a lower loss value, and is the best-performing model. The test accuracy of the ResNet-18 model is lower than that of the HLOA-CNN-BiGRU-Attention model, the test accuracy of the CNN-Transformer and Transformer models is second only to the ResNet-18 model, and the overall trend is stable; but the Transformer model has low accuracy and large fluctuations on the training set and does not achieve good performance in terms of prediction accuracy.

**Figure 21:**
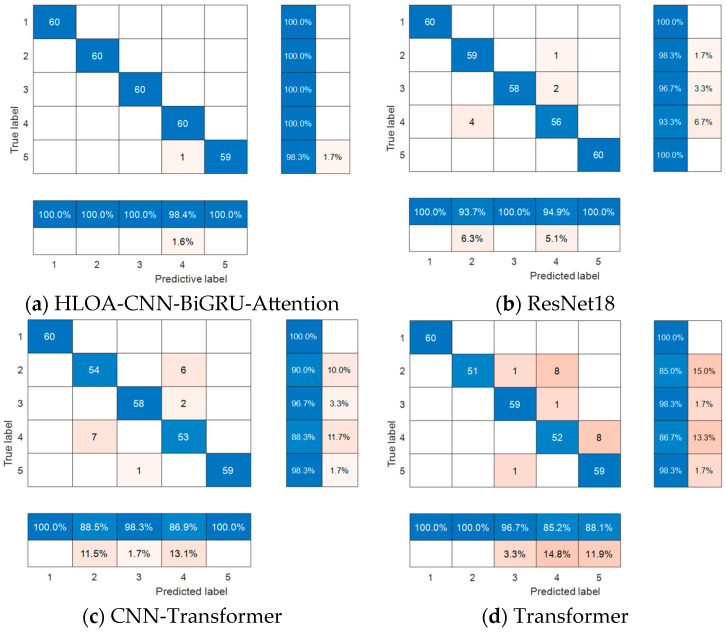
Schematic diagram of the comparison of confusion matrices of various algorithms.

The confusion matrix results of the four different methods on the test set samples are shown in Figure 21. It can be seen from the confusion matrix that each internal leakage condition contains 60 samples, a total of five different internal leakage conditions, and a total of 300 test set samples. The average recognition accuracy of each model for different types of internal leakage of the spring full-lift safety valve is as follows: HLOA-CNN-BiGRU-Attention: 99.7%; ResNet-18: 97.5%; CNN-Transformer: 95.9%; Transformer: 92.9%. It can be seen from the figure that the prediction accuracy of the Transformer and CNN-Transformer models is low, and incomplete prediction occurs in four of the five conditions. ResNet-18 achieved better prediction results among all the comparison models, but the accuracy is still lower than that of the proposed HLOA-CNN-BiGRU-Attention model. Therefore, in the task of identifying different internal leakage states of the spring full-lift safety valve, the proposed HLOA-CNN-BiGRU-Attention method is better than the other three comparison models.

**Figure 22:**
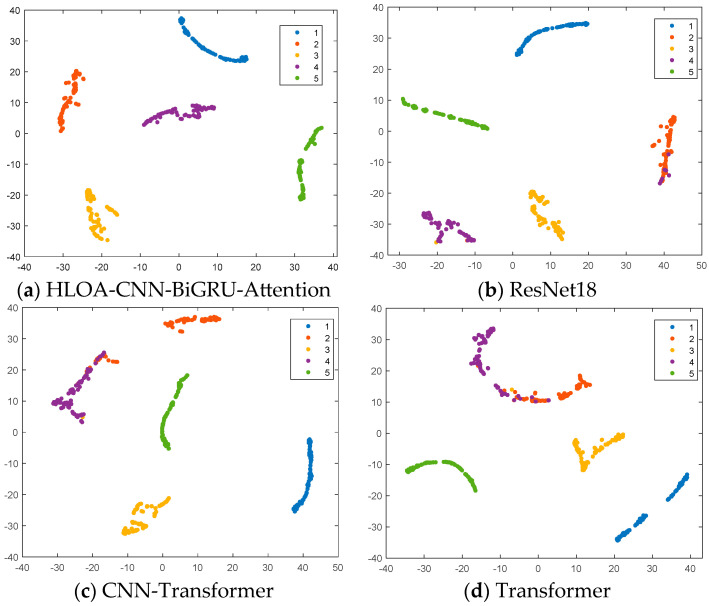
t-SNE diagram of classification of each algorithm.

Figure 22 shows the feature distribution results of the four methods. t-SNE is used in the figure to represent the feature distribution of different internal leakage types of spring full-lift safety valves. Among the four methods, the HLOA-CNN-BiGRU-Attention model performed the best and successfully separated the five categories clearly, reflecting the advantages of the model in dimensionality reduction and maintaining the integrity of data structure. The Transformer and CNN-Transformer methods had poor classification effects, and there was obvious aliasing between categories 2 and 4, indicating that they may be insufficient in capturing high-dimensional features. ResNet-18 also achieved good classification results, but the division between labels 2 and 4 was not clear, and there was a small amount of aliasing. Comparative analysis shows that the HLOA-CNN-BiGRU-Attention method performed best in category visualization and separation effects.

## 5. Discussion of Limitations

(1) This study is aimed at the design of the PN25 DN100 spring full-lift safety valve. Its leakage characteristics (such as the 20 kHz~50 kHz high-frequency energy distribution of the acoustic emission signal) are strongly correlated with the valve structure. Therefore, there may be deviations in the feature extraction of valves of different calibers. For example, the energy of the leakage acoustic emission signal of small-caliber valves (DN25) is more concentrated in the high-frequency band (50 kHz~70 kHz), while the large-caliber valves (DN300) have a wider signal band (10 kHz~60 kHz) due to more complex turbulence. In addition, there are also differences for different types of valves. For example, the sealing structure of other types of valves, such as pressure reducing valves and gate valves, leads to different leakage acoustic emission mechanisms (such as low-frequency vibration, which is the main form of internal leakage in gate valves). Direct migration of the model may reduce the accuracy by 15%~20%. In the future, research will be conducted to solve the universality problem by the fusion of multi-source data and the introduction of domain adaptive algorithms: If there is environmental noise interference in the experiment, such as the vibration noise of compressors, pumps and other equipment may mask the low-frequency signal of the leakage, resulting in the Φ1 mm the accuracy of identifying micro-leakage decreases. In addition, temperature changes will reduce the sensitivity of acoustic emission sensors, and humidity changes may cause signal baseline drift, affecting the stability of the time–frequency diagram. Therefore, in subsequent research, we need to add wavelet thresholds to the STFT preprocessing stage to remove mechanical vibration noise, and combine temperature and pressure sensor data to dynamically adjust the weight of acoustic emission signals through the attention mechanism to improve the diagnostic accuracy of the model in complex environments. The parameter scale of the HLOA-CNN-BiGRU-Attention model used this time is 12.7 M, and it takes about 4.2 h (RTX 3090 single card) after 700 rounds of training. In addition, the parameter search process of the HLOA algorithm adds additional calculations and consumes a lot of resources. In the future, it is necessary to study lightweight models, use MobileNet’s deep separable convolution instead of traditional convolution, compress the parameter scale to 3.2 M, and shorten the edge inference time to 14.8 ms to improve model training efficiency and reduce resource consumption.

(2) Although this study has achieved high-precision identification of the internal leakage state of the safety valve, there are still limitations in the verification and expansion of the model’s generalization ability, as follows: Although the original data set of this study introduces natural variation through cross-batch collection (24-h interval), and the data set covers gradient features from Φ1 mm (small leakage) to Φ2 mm (obvious leakage), the model can accurately distinguish adjacent aperture features, indirectly reflecting the potential generalization ability of “similar unseen faults”. In addition, HLOA is used to optimize the L2 regularization coefficient through global search to suppress the model’s over-learning of noise features, and random pooling is used to fuse the contribution of all high-frequency energy points in the time–frequency graph to reduce misclassification caused by subtle differences in samples. The SK attention module is integrated to improve the generalization design of the model structure through dynamic weighted multi-scale features. However, the existing experimental design only includes five different fault modes, and the sample collection interval is only three batches, which does not cover the influence of sensor drift and environmental temperature and humidity fluctuations on the signal on a long-term time scale. The coverage of fault modes is limited, and the verification of the model’s generalization ability is limited. Therefore, in subsequent research, we will expand the failure mode, collect data regularly to quantify the decay trend of the model accuracy over time, and collect data on safety valves of the same type and different specifications. Through the above improvements, we will further verify the generalization ability of the model and provide more reliable technical support for the real-time diagnosis of safety valve internal leakage.

## 6. Conclusions

This paper uses a high-frequency FPGA data acquisition system to collect acoustic emission signals of different internal leakage states of spring full-lift safety valves. The collected signals are short-time Fourier transformed to obtain a two-dimensional time–frequency graph. The model trained by the improved two-dimensional CNN algorithm is used to intelligently identify the two-dimensional time–frequency graph of different internal leakage states of spring full-lift safety valves. The results show that the internal leakage identification accuracy is 99.7%. The main conclusions of this study are summarized as follows:

(1) Based on the horned lizard optimization algorithm (HLOA) and the improved two-dimensional convolutional neural network (CNN), the bidirectional gated recurrent unit (BiGRU) and SK attention module are synergistically integrated to construct a deep learning internal leakage intelligent recognition model of HLOA-CNN-BiGRU-Attention. This model uses HLOA to optimize the initial learning rate and L2 regularization coefficient of CNN through dynamic switching between local fine-tuning and global exploration, thereby increasing the training accuracy and convergence speed of the model. The LReLU activation function is used in the hybrid convolution kernel neural network to solve the problem of neuron death during negative input, strengthen the characteristic response to weak leakage signals, enhance the nonlinear expression ability, introduce random pooling to fuse all feature elements to prevent overfitting, and improve the robustness of feature extraction. The BiGRU network and SK attention module are synergistically integrated to enhance the ability to extract the temporal and spatial features of the signal while improving the model’s ability to capture multi-scale features, reducing the omission of key information, and solving the parameter optimization problem of high-frequency acoustic emission signals and the limitations of feature extraction.
(2) This study converts the one-dimensional acoustic emission signal into a two-dimensional time–frequency feature image through short-time Fourier transform, effectively retains the signal time–frequency domain information, and provides high-quality input data for the deep learning model. The proposed method can be extended to other complex fault diagnosis scenarios of mechanical equipment based on time series signals or image features. In future research, we will explore lightweight network structures to improve the real-time performance of the model, and at the same time, multi-source sensor data will be used to further improve the robustness of diagnosis.
(3) The average recognition accuracy of the healthy state and internal leakage state of the spring full-lift safety valve using the improved model is 99.7%. The ablation experiment and the mainstream algorithm ResNet-18—97.5%, CNN-Transformer—95.9%, and Transformer—92.9% comparison verifies that the improved convolutional neural network algorithm has advantages in prediction accuracy. Therefore, this method can achieve high-precision fault recognition of different internal leakage states of the spring full-lift safety valve.

## Figures and Tables

**Table 1:** Internal leakage forms of different valve seat sealing surfaces of spring full-lift safety valves.

Working Conditions	Internal Leakage Forms
Working conditions 1	Valve seat sealing surface is not damaged
Working conditions 2	Φ1 mm single semicircular groove
Working conditions 3	Φ2 mm single semicircular groove
Working conditions 4	Φ1 mm double semicircular groove
Working conditions 5	Φ2 mm double semicircular groove

**Table 2:** Data set information under different forms of internal leakage.

Internal Leakage Form	Number of Training Sets	Number of Test Sets	Total Sample Size	Sample Proportion	Label
Valve seat sealing surface is not damaged	140	60	200	20%	1
Φ1 mm single semicircular groove	140	60	200	20%	2
Φ2 mm single semicircular groove	140	60	200	20%	3
Φ1 mm double semicircular groove	140	60	200	20%	4
Φ2 mm double semicircular groove	140	60	200	20%	5

**Table 3:** Confusion matrix calculation precision, recall, and F1 score.

Label	Number of Samples	TP	FP	FN	Accuracy $\mathrm{Accuracy}=\frac{\mathrm{TP}}{\mathrm{TP}\;+\;\mathrm{FP}}$	Recall $\mathrm{Recall}=\frac{\mathrm{TP}}{\mathrm{TP}\;+\;\mathrm{FN}}$	F1 Score $F1\;\mathrm{score}\;=\;2\frac{\mathrm{Accuracy}\;\times \;\mathrm{Recall}}{\mathrm{Accuracy}\;+\;\mathrm{Recall}}$
1	60	60	0	0	1.00	1.00	1.00
2	60	60	0	0	1.00	1.00	1.00
3	60	60	0	0	1.00	1.00	1.00
4	60	60	1	0	0.984	1.00	0.992
5	60	59	0	1	1.00	0.983	0.991

## Data Availability

The original contributions presented in this study are included in the article. Further inquiries can be directed to the corresponding author.
